# Cognitive-Motor Interference during Walking in Older Adults with Probable Mild Cognitive Impairment

**DOI:** 10.3389/fnagi.2017.00350

**Published:** 2017-12-11

**Authors:** Thomas J. Klotzbier, Nadja Schott

**Affiliations:** Department of Sport and Exercise Science, Institute for Sport and Exercise Science, University of Stuttgart, Stuttgart, Germany

**Keywords:** dual task costs, Trail-Walking Test, visuo-spatial working memory, executive attention network, cognitive reserve, reliability

## Abstract

Although several studies have shown that dual-tasking (DT) mobility is impaired in Alzheimer's disease, studies on the effects of DT conditions in probable Mild Cognitive Impairment (pMCI) have not yielded unequivocal results. The objectives of the study were to (1) examine the effect of a concurrent task on a complex walking task in adults with cognitive impairment; and (2) determine whether the effect varied with different difficulty levels of the concurrent task. Furthermore, the study was designed to evaluate the Trail-Walking Test (TWT) as a potential detection tool for MCI. We examined DT performance in 42 young adults (mean age 23.9 ± 1.98), and 43 older adults (mean age 68.2 ± 6.42). The MoCA was used to stratify the subjects into those with and without pMCI. DT was assessed using the TWT: participants completed 5 trials each of walking along a fixed pathway, stepping on targets with increasing sequential numbers (i.e., 1-2-…-15), and increasing sequential numbers and letters (i.e., 1-A-2-B-3-…-8). Motor and cognitive DT effects (DTE) were calculated for each task. ROC curves were used to distinguish younger and healthy older adults from older adults with pMCI. The TWT showed excellent test-retest reliability across all conditions and groups (ICC : 0.83–0.97). SEM% was also low (<11%) as was the MDC95% (<30%). Within the DT conditions, the pMCI group showed significantly longer durations for all tasks regardless of the cognitive load compared to the younger and the healthy older adults. The motor DTEs were greatest for the complex condition in older adults with pMCI more so than in comparison with younger and healthy older adults. ROC analyses confirmed that only the tasks with higher cognitive load could differentiate older adults with pMCI from controls (area under the curve >0.7, *p* < 0.05). The TWT is a reliable DT mobility measure in people with pMCI. However, the condition with high cognitive load is more sensitive than the condition with low cognitive load in identifying pMCI. The TWT-3 thus could serve as a screening tool for early detection of individuals with pMCI. Future studies need to determine the neural correlates for cognitive-motor interference in older adults with pMCI.

## Introduction

Cognitive impairments such as Mild Cognitive Impairment (MCI) and dementia are the most important public health challenges of the twenty-first century. As age is the key risk factor the World Health Organization (WHO) predicts that 115.4 million people will suffer from dementia by 2050. MCI has been designated as the transitional stage from mild impairment to severe dementia (Janoutov et al., 2015). Although the main symptoms of MCI are memory impairment and executive dysfunction, motor dysfunction (including gait impairment) has been previously described as a common characteristic in participants with cognitive impairments (Verghese et al., 2008).

The search for useful markers in MCI, especially motor markers, is a promising new research field (Pettersson et al., 2005; Aggarwal et al., 2006; Montero-Odasso et al., 2009; Beauchet et al., 2013; Verghese et al., 2013). Not least because there has been growing interest in early detection and effective strategies for prevention (Barnes and Yaffe, 2011). As cognition and gait are thought to be strongly linked, the cognitive-motor interference (CMI) approach using dual task (DT) walking, may be a new methodological approach for the evaluation of brain function in MCI (Montero-Odasso et al., 2014). CMI refers to the phenomenon in which carrying-out simultaneously a motor and a cognitive task interferes with the performance of one or both tasks (Schott et al., 2016). Lundin-Olsson et al. (1997) were the first who showed that those who had to “stop” during a conversation had a greater risk of falling. She and her colleagues were thus able to demonstrate the effect that cognitive load has on gait. Gross-motor performance such as functional goal-oriented locomotion is thus not a merely automatic process, but requires higher-level cognitive input, highlighting the relationship existing between cognitive function and walking (Schott et al., 2016). This interdependence between gait and cognition in older people is demonstrated with the fact that slow gait performance is more prevalent in people with cognitive impairment and dementia (Camicioli et al., 1998; van Iersel et al., 2004; Allan et al., 2005; Pettersson et al., 2005; Holtzer et al., 2006; Montero-Odasso et al., 2009). In subsequent years, it has been established that the effect of dual tasking on gait velocity is related to impairments in executive function (EF) and attention (Sheridan et al., 2003; Camicioli et al., 2006).

Although significant dual task effects have been found in dementia (Manckoundia et al., 2006; Allali et al., 2007; Pettersson et al., 2007) studies on the effect of dual tasking in MCI have not yielded unambiguously results (Bahureksa et al., 2017). Montero-Odasso et al. (2009) revealed that dual task conditions show the strongest association with gait slowing. Their findings suggest that the control of gait is associated with decline in working memory in people with MCI. However, a comparison between MCI patients and healthy subjects was not possible because they did not include a control group. Pettersson et al. (2007) investigated the influence of cognition on motor function and evaluated the reliability for the dual task test “Talking While Walking.” Subjects with Alzheimer's disease and MCI produce lower walking speeds; furthermore, they exhibited greater time change between single and dual task compared with healthy controls. The authors conclude that decreased walking speed during single- and dual task performance may be an early symptom in Alzheimer's disease. In a previous study, the authors indeed were able to show that motor function seems not to be affected in MCI (Pettersson et al., 2005). Tseng et al. (2014) were able to show that participants with MCI manifested a more slowing gait than age- and education-matched cognitively normal adults under dual task conditions. The largest differences were observed during test of working and episodic memory. The authors concluded that the outcome of a dual task assessment shows promise as a potential marker for early detection of MCI. Montero-Odasso et al. (2014) described that participants with MCI have poor gait performance particularly under dual tasking. Their findings suggest that dual task tests can help to differentiate between different subtypes of MCI, describing DT results as a motor signature in MCI. Amboni et al. (2012) report similar results. They were able to show that dysfunction on gait in Parkinsonian patients with and without MCI is highly sensitive to dual-task conditions. In contrast, Nascimbeni et al. (2015) suggest that the use of a dual task paradigms do not improve the early detection of MCI. They used three different cognitive tasks (counting backwards, short story recall and a phonemic fluency task) to investigate the motor-cognitive interference in a sample of MCI patients and a group of matched healthy controls. Both groups showed an effect of dual task interference on gait independently of the kind of cognitive task, so there were no significant group differences visible while dual tasking.

However, the consistent prediction of falls or detection of MCI by dual task testing remains difficult. Nascimbeni et al. (2015) note that in most studies the effect of dual task interference on the cognitive tasks was not analyzed. Therefore, helpful insights into the possible preferential allocation of limited attentional resources in MCI patients carrying out a cognitive task during walking are still missing. Furthermore, the tasks of daily living require not only going on straight stretches. Rather obstacles have to be avoided, people must be dodged and streets have to be navigated through (Schott, 2015). Compared to straight-path walking, different cognitive functions are addressed during curved-path or zig-zag-walking. While the simple walking task on straight stretches can be mostly solved by information processing, cognitive flexibility and set-shifting processes uniquely contribute to how individuals navigate through curved paths. Hence, the measure of change of direction walking provides different and meaningful information about daily life walking ability than walking on straight pathways alone (Lowry et al., 2012). Salkovic et al. (2017) were able to demonstrate that a priorization of resource allocation is influenced by the walking situation. According to their results older adults with poor cognitive flexibility exhibit a tendency to riskier walking behavior during more complex walking situations, compared to older adults with good cognitive flexibility.

An elegant solution was first proposed by Alexander et al. (2005), and further developed by Yamada and Ichihashi (2010) and Schott (2015). They converted the fall risk associated standard neuropsychological test (Trail-Making Test A) with increasing cognitive load into a mobility task and determined the usefulness of this Trail-Walking Test (TWT) for predicting falls in community-dwelling elderly individuals. However, with this test an account on the pure motor component, in terms of a tracking task cannot be made. Thus, the calculation of actual DT interference is impossible (Schott, 2015). For this reason Schott (2015) further developed and expanded the TWT and included a purely motor task as well as the TMT B. She demonstrated that the TWT is a feasible, reliable and valid tool to discriminate between older non-fallers and fallers.

This test, which includes walking in a more ecological valid dual task, should help to better understand the relationship between cognitive function and gait in people with MCI. Therefore, the present study was designed to evaluate the TWT as a potential detection tool for MCI. We hypothesized that overall, individuals with probable MCI (pMCI) exhibit proportionally greater dual task effects (DTE) under more complex, attention demanding motor and cognitive tasks relative to younger and older adults with and without pMCI (see also Schott et al., 2016). Therefore, higher motor interference indicate requirement of greater attentional resources for the cognitive task, under DT conditions. Tasks showing higher cognitive interference indicate prioritization of the motor task under the respective DT condition and lower cognitive cost would indicate prioritization of the cognitive task under the respective DT condition (for a more detailed explanation see Plummer and Eskes, 2015). Based on the difficulties of older adults with pMCI across core domains of EF, including working memory (WM) (averaged over visuospatial and verbal), inhibitory control, cognitive flexibility, and executive attention, we predicted that older adults with pMCI would perform more poorly than the control group on the TWT-3 (high cognitive load) and to a lesser extent on the TWT-2 (reduced cognitive load) (Walshe et al., 2015; Schott et al., 2016).

## Materials and methods

### Participants

Forty-two healthy young adults (18 female; *M* = 23.9, *SD* = 1.98, age range 20–28) and 43 older adults (21 female; *M* = 68.2, *SD* = 6.42, age range 60–81) participants volunteered to take part in the experiment. Young subjects were recruited from the university community. Older participants were all community dwelling and were recruited through word of mouth and contacts at social or leisure clubs. We applied the following inclusion criteria when recruiting older participants: 60 years of age or older, reported normal or corrected-to-normal vision and hearing, ability to walk independently, and ability to follow instructions for testing. Subjects were excluded if they had musculoskeletal disorders such as arthrosis impairing posture or gait, central or peripheral neurological diseases (e.g., previous stroke, Parkinson's disease), recent acute illness or surgery, psychiatric disorders and/or the use of psychiatric drugs that may affect cognitive performance (Nascimbeni et al., 2015).

PMCI is defined by objective evidence of impairment in one or more cognitive domains, typically memory problems (Albert et al., 2011; American Psychiatric Association, 2013). In this study, we assessed cognitive function with the Montreal Cognitive Assessment (MoCA; score range: 0–30; Nasreddine et al., 2005). This tool has shown to be sensitive to mild cognitive deficits when applied in cognitively intact older adults (Duffin et al., 2012; Kenny et al., 2013). It includes measures of EF, language, memory, attention, orientation, calculation, and visuospatial ability. The age- and education adjusted MoCA measure^1^ (Santangelo et al., 2015), was used to categorize the 43 older participants into two groups: cognitively intact older adults (MoCA > 25; *n* = 24) or pMCI (MoCA ≤ 25; *n* = 19). This cutoff was found to be the optimal cutoff point for a diagnosis of pMCI with high specificity as well as high reliability (Nasreddine et al., 2005). While Lonie et al. (2009) suggested that, of the existing tools used to screen for global cognitive status, the MoCA is among the most optimal for the detection of MCI, Lister et al. (2016) pointed out, that it is important to acknowledge that participants with low scores on the MoCA could have had neurological disorders other than MCI, which were not reported in the preliminary examination.

All participants were informed of the nature and aim of the study, and signed a consent form. All procedures were in accordance to the Declaration of Helsinki with ethical standards, legal requirements and international norms. An internal ethics committee at the University of Stuttgart approved the study. Furthermore, we have ethics approval from the Wilfried Laurier University, Waterloo, Canada for a study (Motor-cognitive interference in dual tasks: allocation of resources in Parkinson Disease patients) using this protocol and additional assessments (REB # 4791 Project, “Motor-cognitive interference in dual tasks: allocation of resources in Parkinson Disease patients” REB Clearance Issued: February 19, 2016).

### Measures

#### Trail-making-test (Reitan, 1955)

The Trail-Making-Test (TMT; Reitan, 1955) was used to assess executive function. Originally, the paper-and-pencil test is mainly applied in the diagnostics of Alzheimer's disease, and consists of two parts. During Part A subjects are instructed to connect encircled numbers (1–25) in ascending order randomly distributed on a white sheet of paper. The TMT-A assesses attention, visual scanning, motor speed and coordination. During part B (TMT-B), participants are asked to connect randomly positioned circles alternating between ascending numbers (1–13) and letters (A to L) (1-A-2-B- etc.). The TMT-B assesses mental flexibility and working memory in addition to the abilities assessed by part A. The TMT is a reliable and valid measure (Bowie and Harvey, 2006). TMT-A and TMT-B include the simultaneous (dual-task) performance of a cognitive and in this case a fine motor control task. Additionally, we included a motor speed condition (TMT motor speed) as single task condition: subjects trace over a dotted line connecting circles on the page (trail of the same length compared to TMT A), in order to test their ability to adapt movement accuracy to spatial constraints based on incoming visual feedback with temporal pressure (see also Schott et al., 2016). Participants were instructed to trace as accurately and as quickly as possible (arrows indicated tracing direction). Each condition is preceded by a short practice trial. Errors are corrected when they occur, thus increasing the time taken to complete the test. The trials were timed using a stopwatch to the nearest 0.01 s. Due to the longer total trail length of TMT B compared to TMT A (Gaudino et al., 1995) and TMT motor speed we report the velocity (cm/s) instead of the total duration.

#### Trail-walking-test (Schott, 2015)

**Figure 1:**
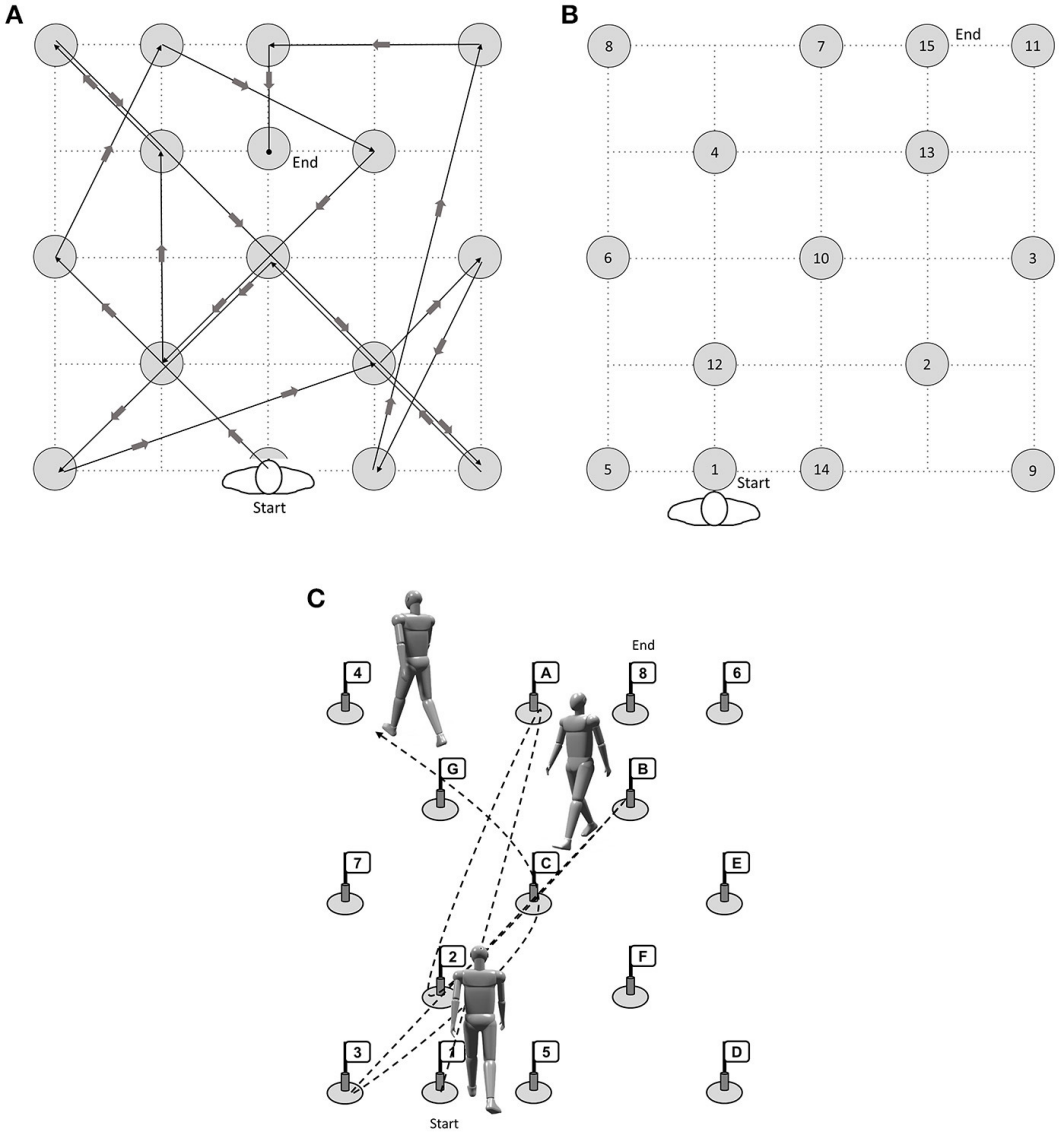
**(A)** Condition 1 (motor condition), **(B)** Condition 2 (attention, visual scanning, information processing speed) and **(C)** Condition 3 (cognitive flexibility, inhibition, working memory) of the Trail-Walking Test (length 41 m) (modified after Schott, 2015).

Participants also performed a novel walking task modeled after the TMT. For the Trail-Walking Test (TWT; Schott, 2015; Schott et al., 2016), cones with flags are placed randomly at each of 15 positions in a 16-m^2^ area (4 × 4 m). A 30-cm diameter circle was drawn around each cone. The participants are instructed to follow a fixed pathway (TWT-1, see Figure 1A), step on targets with increasing sequential numbers (i.e., 1-2-3; TWT-2, see Figure 1B), and increasing sequential numbers and letters (i.e., 1-A-2-B-3-C; TWT-3; see Figure 1C). Passage was considered to be successful when the participant didn't knock over a cone, stepped on the circle, and didn't walk in the wrong direction. Participants were instructed to move from one flag to the next one in ascending order as quickly, but as accurately as possible. However, no priority was given to one domain or the other. Only in condition 1, floor markings were used to show participants which way to follow. The trials were timed using a stopwatch to the nearest 0.01 s following a standard procedure. Mistakes were recorded. Each condition was performed five times.

Additionally, participants were instructed to walk along a 10 m walkway that had a 2 m buffer space at both ends for acceleration and deceleration. The time to walk 10 m was measured, and gait speed was expressed in meter per second.

#### Sociodemographic information, health characteristics, falls and falls-associated self-efficacy

Sociodemographic and health characteristics included age (20–30, 60–81), gender, education, medication, BMI, and physical activity. Height and weight of the participants were measured, and body mass index (BMI, kg/m^2^) was calculated. Physical activity was measured using the Instrument for the assessment of middle-aged and older adults' physical activity (German-PAQ-50+; Huy and Schneider, 2008). Falls-associated self-efficacy was assessed by the German short version of the Activities specific Balance Confidence (ABC-D6, Schott, 2014). Participants provided a history of falls occurring in the 6 months prior to enrollment in the study. A fall was defined as an event resulting in an individual inadvertently coming to rest on the ground or at a lower level, not due to a major intrinsic event (e.g., loss of consciousness) or overwhelming hazard (Finlayson and Peterson, 2010).

### Procedure

The testing was conducted in a quiet environment to complete the testing battery, which could be administered in one session over the course of a day. After obtaining consent and collecting the socio-demographic, anthropometric, physical activity, and falls-related data, the older participants completed the Montreal Cognitive Assessment (MoCA).

To ensure that participants understood the concept of the Trail-Making-Test, we started with examples of the paper-pencil version, followed by the test version. Next, participants walked at their fast, but still comfortable speed along a 10 m walkway. Then they completed the Trail-Walking-Test in random order with directions provided using a standard script, walking five times during each condition.

### Data analysis

Statistical analyses were implemented on SPSS v.24 (SPSS, Chicago, IL) and MedCalc version 17. We first explored dependent variables to examine missing data points, normality of distributions (tested by Kolmogorov–Smirnov tests), and presence of outliers (defined by the Explore command of SPSS v.24). An alpha level of 0.05 was used for all statistical tests.

Potential baseline group differences for continuous variables (i.e., age, height, weight, BMI, physical activity, ABC, falls) were assessed using ANOVAs, and categorical demographic variables (i.e., gender, weight category) were compared by chi-square test.

For assessing the reliability of each of the conditions of the Trail-Walking Test across the three groups, the two-way mixed model (type: consistency) intraclass correlation coefficient (ICC) was used and the between-trial reliability (five trials) for time was calculated. An ICC of < 0.69 represents poor reliability; 0.70 ≤ ICC ≤ 0.79 represents fair reliability, 0.80 ≤ ICC ≤ 0.89 represents fair reliability and ICC > 0.90 represents excellent reliability (Fleiss, 1999).

Absolute test–retest reliability was evaluated by the estimation of the standard error of measurement (SEM) and the Minimum Detectable Change at a 95% confidence interval (MDC_95_). The SEM is a measure of the precision of individual scores on a test (Weir, 2005). The SEM was estimated using the square root of the within-subjects error variance (SEM = SD × SQT(1-ICC)). To define the smallest change that indicates a real (clinical) improvement or a deterioration for a single individual, we used the smallest real difference, MDC_95_ (1.96 × SEM × SQT(2)). Both measures were also expressed as a percentage of the mean (i.e., SEM% and MDC_95%_), to produce unitless indicators and allow for comparisons. A SEM% (SEM/mean)^*^100) smaller than 10% of the mean test and retest scores were considered to indicate excellent absolute reliability (Atkinson and Nevill, 1998).

The Pearson product-moment correlation (*r*) was used to examine the degree of association between durations for the TMT, TWT, DTEs and the participant characteristics. To examine the effect of the different cognitive task conditions on the motor tasks, each variable was analyzed using a 3 × 3 repeated measure analysis of variance (ANCOVA), adjusted for demographic variables, if appropriate, with task conditions as the within-subjects factor (motor task only, numbers condition, numbers & letters condition) and one between-subjects factor (younger adults, older adults with and without pMCI) (Schott et al., 2016).

Dual task effects (DTE): The effect of dual tasking on both motor and cognitive parameters was assessed by comparing the absolute values for all cognitive and motor parameters between single- and DT-conditions. To compare the motor and cognitive function across the different DT conditions, the motor and cognitive DTEs calculated according to the common formula (Plummer and Eskes, 2015):

$$\begin{array}{l} \text{DTE }(\%)\;=\frac{-(\text{Dual task time}-\text{Single task time})}{\text{Single task time}}*100 \end{array}$$

Here, the negative sign is included because higher values for the duration for these tasks represent worse performances. Therefore, negative DTE values indicate that performance deteriorated in the dual-task relative to the single- task (i.e., dual-task cost), whereas positive DTE values indicate a relative improvement in performance in the dual-task (i.e., dual- task benefit) (Plummer and Eskes, 2015, p. 3).

Because motor performance can deteriorate in one or both of the activities performed simultaneously when they exceed the available attentional resources, it is important to examine change in both activities; thus, we examined motor and cognitive DTEs (Schott et al., 2016). The motor and cognitive interference effects across the two DT conditions for gross motor control tasks were compared using a 3 (group) × 2 (condition: low and high cognitive load) × 2 (CMI: cognitive vs. motor interference) repeated measures ANCOVA, adjusted for demographic variables, if appropriate (see also Schott et al., 2016).

Effect size for all ANOVAs was reported using partial eta squared (η_*p*_^2^), with a small effect defined as 0.01, a medium effect as 0.06, and a large effect as 0.14 (Cohen, 1988). Repeated measures sphericity issues were addressed with the Greenhouse Geisser correction. When ANOVAs were statistically significant, *post-hoc* comparisons were performed using the Bonferroni correction. The level of significance for *post-hoc* comparisons was set at 0.05 (Tabachnick and Fidell, 2013).

The receiver operating characteristics (ROC) curves were also used to further determine whether the single-task and dual-task assessments were useful in distinguishing younger and healthy older adults from older adults with pMCI. The area under the ROC curve (AUC) was also calculated. The cutoff score was determined by visual inspection of the ROC plot as well as the Youden's index (sensitivity+(specificity-1) (Hilden and Glasziou, 1996).

## Results

### Characteristics of the study population

Table 1 summarizes the demographic data including BMI, educational history, current medication, physical activity, exercise, functional capacity, falls-associated self-efficacy, and the number of subjects, who experienced a fall in the last 6 months.

**Table 1:** Demographic and general assessment data of the young and the older groups with and without cognitive impairment (*n* = 85).

	**Young adults**	**Healthy older adults MoCA > 25**	**pMCI MoCA ≤ 25**	**Stat. analysis**
	**(*n* = 42)**	**(*n* = 24)**	**(*n* = 19)**	
Sex	24M, 18F	11M, 13F	11M, 8F	${\mathrm{CHI}}_{\left(2\right)}^{2}$ = 0.93
Age (years)	23.9 (1.98)	67.5 (6.88)^ŧ^	69.1 (5.86)^ŧ^	*F*_(2, 82)_ = 914^**^, η^2^*_*p*_* = 0.957
BMI (kg/m^2^)	22.9 (2.18)	24.6 (3.83)	26.0 (4.09)^ŧ^^,^^ᵻ^	*F*_(2, 81)_ = 6.62^**^, η^2^*_*p*_* = 0.140
MOCA adj.	NA	27.0 (1.76)	23.5 (2.35)	*t*_(41)_ = 5.69^**^, *d* = 1.78
Education (years)	13.2 (2.02)	11.3 (2.64)^ŧ^	12.2 (3.14)	*F*_(2, 82)_ = 5.01^**^, η^2^*_*p*_* = 0.109
Medication (n)	0.10 (0.30)	0.71 (0.81)^ŧ^	1.16 (0.96)^ŧ^	*F*_(2, 82)_ = 18.9^**^, η^2^*_*p*_* = 0.315
**TMT**				
Motor speed (cm/s)	16.2 (4.13)	10.5 (2.90)^ŧ^	8.65 (3.08)^ŧ^	*F*_(2, 82)_ = 35.8^**^, η^2^*_*p*_* = 0.466
A (cm/s)	9.76 (2.71)	5.75 (1.40)^ŧ^	4.38 (0.87)^ŧ^	*F*_(2, 82)_ = 54.2^**^, η^2^*_*p*_* = 0.569
B (cm/s)	5.21 (1.58)	3.20 (1.17)^ŧ^^,^^ᵻ^	1.97 (0.57)^ŧ^^,^^ᵻ^	*F*_(2, 82)_ = 45.4^**^, η^2^*_*p*_* = 0.526
PAQ50+ (h/week)	43.7 (21.8)	47.9 (22.5)	53.5 (30.3)	*F*_(2, 82)_ = 1.11, η^2^*_*p*_* = 0.026
Exercise in a club (min/week)	289 (218)	118 (205)^ŧ^	109 (102)^ŧ^	*F*_(2, 82)_ = 8.67^**^, η^2^*_*p*_* = 0.174
**10 m-GAIT**				
Velocity (m/s)	2.16 (0.40)	1.95 (0.42)	1.87 (0.56)	*F*_(2, 82)_ = 3.49^*^, η^2^*_*p*_* = 0.078
Steps (n)	11.9 (1.40)	13.1 (1.96)^ŧ^	13.0 (2.05)	*F*_(2, 81)_ = 4.59^*^, η^2^*_*p*_* = 0.102
ABC (%)	92.4 (7.46)	85.0 (16.2)	80.0 (15.7)^ŧ^	*F*_(2, 82)_ = 4.88^**^, η^2^*_*p*_* = 0.106
≥1 fall in the last 6 months (n)	0.17 (0.38)	0.25 (0.44)	0.37 (0.50)	*F*_(2, 82)_ = 1.50, η^2^*_*p*_* = 0.035

* *p < 0.05*;

** *p < 0.01*;

ŧ *Significant difference from the younger group (p < 0.05)*;

ᵻ *Significant difference from the older adults with a MoCa > 25 (p < 0.05)*.

As might be expected, the ones with pMCI scored worse on BMI, education, medication, exercise, gait velocity, and falls-associated self-efficacy, compared to younger adults, but not compared to older adults with a MoCA higher than 25. Chi-Square analysis indicated only a significant difference between groups for BMI, $\chi_{\left(4\right)}^{2}$ = 9.64, *p* = 0.047 with older adults with pMCI exhibiting a higher number of overweight and obese subjects (52.6%) compared to younger adults (19.0%) and healthy older adults (43.5%).

### Trail-making test

The overall mean velocities of the modified Trail-Making Test in single and dual conditions by group are shown in Table 1. Scores of raw were normally distributed in all three participant groups, *p* > 0.05. When demographic variables (age, education, MoCA, and sex) were related to TMT velocities, age (*r* = −0.705 to −0.743, *p* < 0.001), education (*r* = 0.267 −0.271, *p* < 0.05), and MoCA (*r* = 0.370 −0.427, *p* < 0.05) were significantly correlated with TMT motor speed, TMT A, and TMT B. Time to completion did not differ by sex for either TMT motor speed, TMT A or TMT B.

A 3 (group: young adults vs. older adults, MoCA > 25 vs. older adults, MoCA ≤ 25) × 3 (condition: motor task only, numbers, numbers & letters) ANCOVA with repeated measures adjusted for age on the velocities of the TMT was conducted, which showed a significant effect of condition, *F*_(1.36, 109)_ = 7.94, *p* = 0.002, η_*p*_^2^ = 0.090. *Post-hoc* analysis indicated that all three conditions of the TMT were significantly different from each other for the total group as well as for the three performance groups (*p* < 0.001). No interaction between condition and group was observed.

### Trail-walking test

Relative and absolute reliability statistics (ICC, SEM, MDC_95_) are shown in Table 2. The between-trial reliability was good to excellent for all conditions and all groups with ICC values ranging from 0.83 to 0.97. In the total group, SEM ranged 1.16–4.11 s. SEM% was low for almost all variables and all groups (4.2–8.3%). In 98% of observations, a SEM% ≤ 10% was obtained. SEM ranged 1.06–3.18 s in der group of younger adults, 1.28–3.95 s in the group of older adults without pMCI, and 1.44–7.76 s in the group of older adults with pMCI. Subgroup comparison revealed a higher number of variables below the threshold (SEM% ≤ 10%) in the younger group and the group without pMCI (100%) compared to the group with pMCI (95%). For the total group, MDC_95_ ranged 3.2–11.5 s for durations. MDC_95%_ ranged 11.5–23.2%, and was therefore ≤30% for the whole sample.

**Table 2:** Results for relative (ICC) and absolute (SEM) inter-trial reliability, and MDC_95_ for each condition of the TWT.

	**Young adults**			**Older adults MoCA** > **25**			**Older adults MoCA** ≤ **25**		
	**ICC (95% CI)**	**SEM/SEM (%)**	**MDC_95_/MDC_95_%**	**ICC (95% CI)**	**SEM/SEM (%)**	**MDC_95_/MDC_95_%**	**ICC (95% CI)**	**SEM/SEM (%)**	**MDC_95_/MDC_95_%**
TWT-1	0.901 (0.85–0.94)	1.06/4.62	2.95/12.9	0.967 (0.94–0.98)	1.28/4.00	3.57/11.2	0.968 (0.94–0.99)	1.44/4.15	4.02/11.6
TWT-2	0.908 (0.86–0.95)	1.50/4.94	4.18/13.8	0.884 (0.79–0.94)	2.57/5.79	7.19/16.2	0.892 (0.79–0.95)	4.65/8.81	13.0/24.6
TWT-3	0.864 (0.79–0.92)	3.18/8.72	8.88/24.4	0.869 (0.76–0.94)	3.95/7.24	11.0/20.2	0.827 (0.66–0.93)	7.76/10.8	21.7/30.1

When demographic variables (age, education, ABC-D6, MoCA, and sex) were related to TWT durations, age (*r* = −0.761 to −0.824, *p* < 0.001), education (*r* = −0.191 −0.259, *p* < 0.10), ABC-D6 (*r* = −0.240 −0.587, *p* < 0.01), and MoCA (*r* = −0.410 −0.530, *p* < 0.01) were significantly correlated with TWT-1, TWT-2, and TWT-3. Differences for sex were only found for the TWT-1, [*t*_(60.2)_ = −2.55, *p* = 0.017, *d* = −0.66], with men walking faster compared to women.

**Figure 2:**
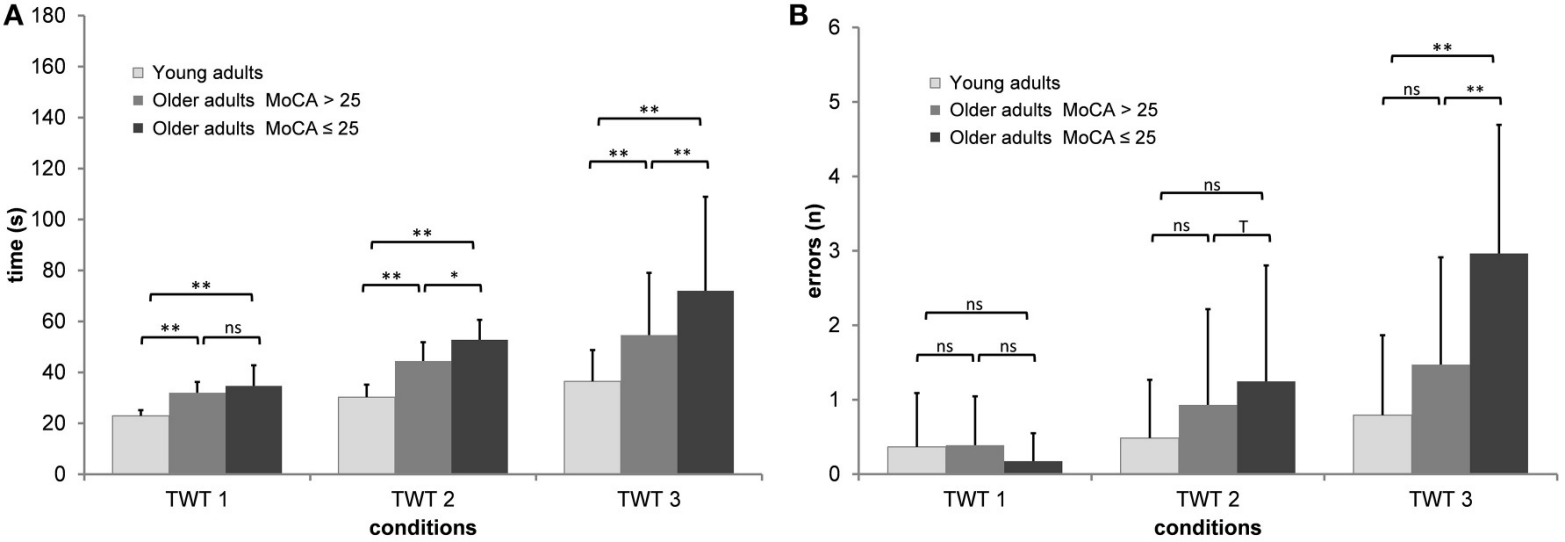
Means and standard deviation for **(A)** durations and **(B)** number of errors by group, and condition of the Trail-Walking-Test (^**^p < 0.01; ^*^p < 0.05; ^T^*p* < 0.10; ns, non-significant).

A 3 (group) × 3 (condition: motor task only, numbers, numbers, and letters) × 5 (trials) ANCOVA with repeated measures adjusted for age and falls-associated self-efficacy on the times of the TWT was conducted, which showed a significant interaction of condition × group, *F*_(3, 56, 142)_ = 14.6, *p* < 0.001, η_*p*_^2^ = 0.267. This interaction showed that older adults had longer durations for the task with higher cognitive loads than younger adults; in the task with the highest cognitive load older adults without pMCI outperformed the adults with pMCI (see Figure 2A).

*Post-hoc* analysis indicated that all three conditions were significantly different from each other for the total group as well as for almost all group comparisons (*p* < 0.05). While there were almost no differences for the motor speed condition across trials, especially participants with pMCI improved their performance times for the conditions with low and high cognitive load across trials, *F*_(9.70, 388)_ = 1.64, *p* = 0.095, η_*p*_^2^ = 0.039.

A 3 (group) × 3 (condition: motor task only, numbers, numbers, and letters) × 5 (trials) ANCOVA with repeated measures adjusted for age and education on the errors of the TWT was conducted, which showed a significant interaction of condition × group, *F*_(4, 160)_ = 6.94, *p* < 0.001, η_*p*_^2^ = 0.148. This interaction showed that older adults produced more errors for the task with higher cognitive loads than younger adults; in the task with the highest cognitive load older adults without pMCI outperformed the adults with pMCI (see Figure 2B). *Post-hoc* analysis indicated that only TWT-2 and TWT-3 were significantly different from each other for the total group. Only for the TWT-3 we observed significant differences between older adults with pMCI and the two other groups (*p* < 0.01).

**Figure 3:**
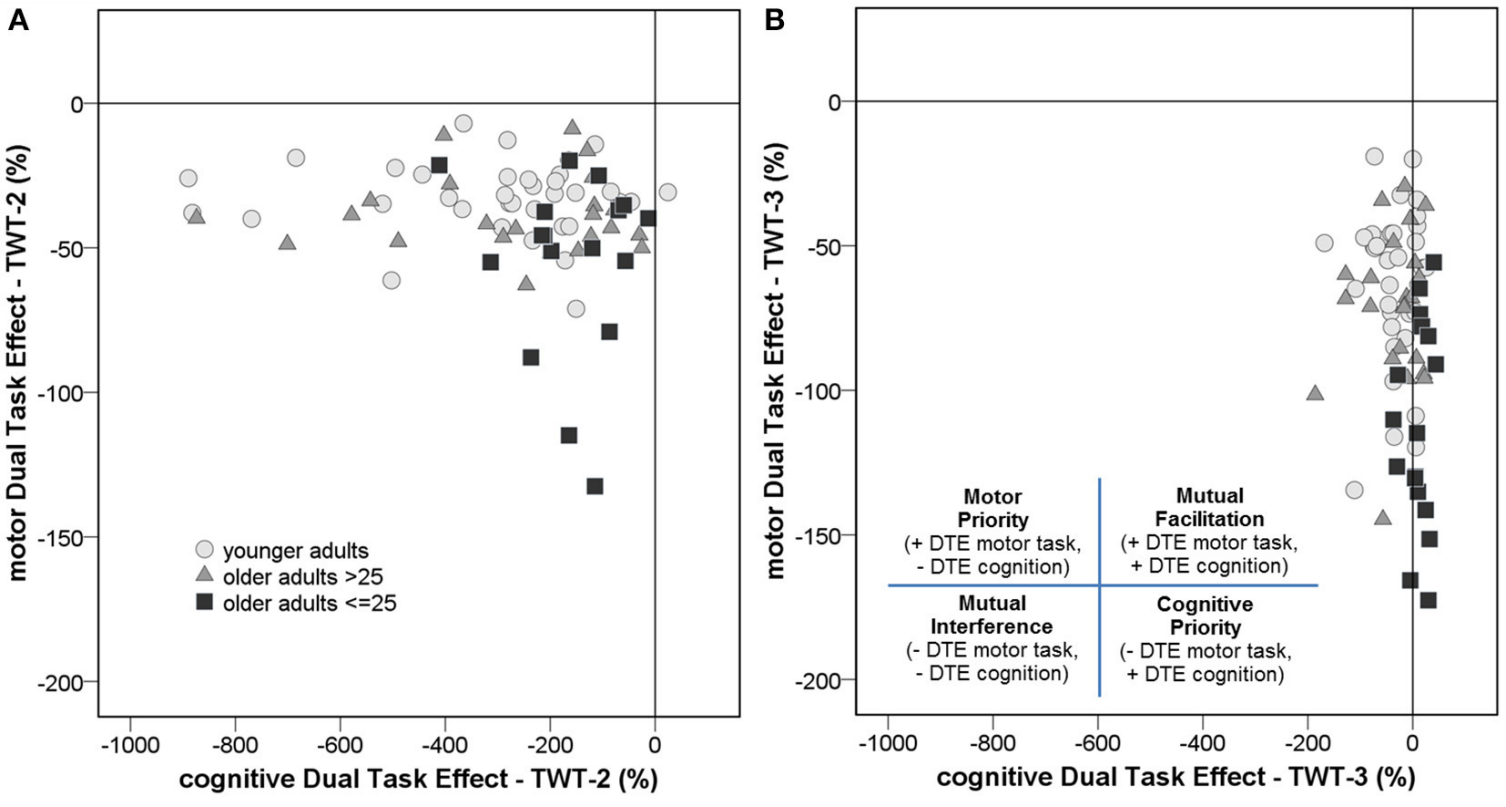
An examination of the dual-task effects of the cognitive and motor conditions on the **(A)** TWT-2 and the **(B)** TWT-3 by group reveals that the majority of individuals either experienced mutual interference, where both motor and cognitive performance declined under dual-task conditions, or prioritized cognition, such that motor performance decreased but cognitive performance increased under dual-task conditions.

**Figure 4:**
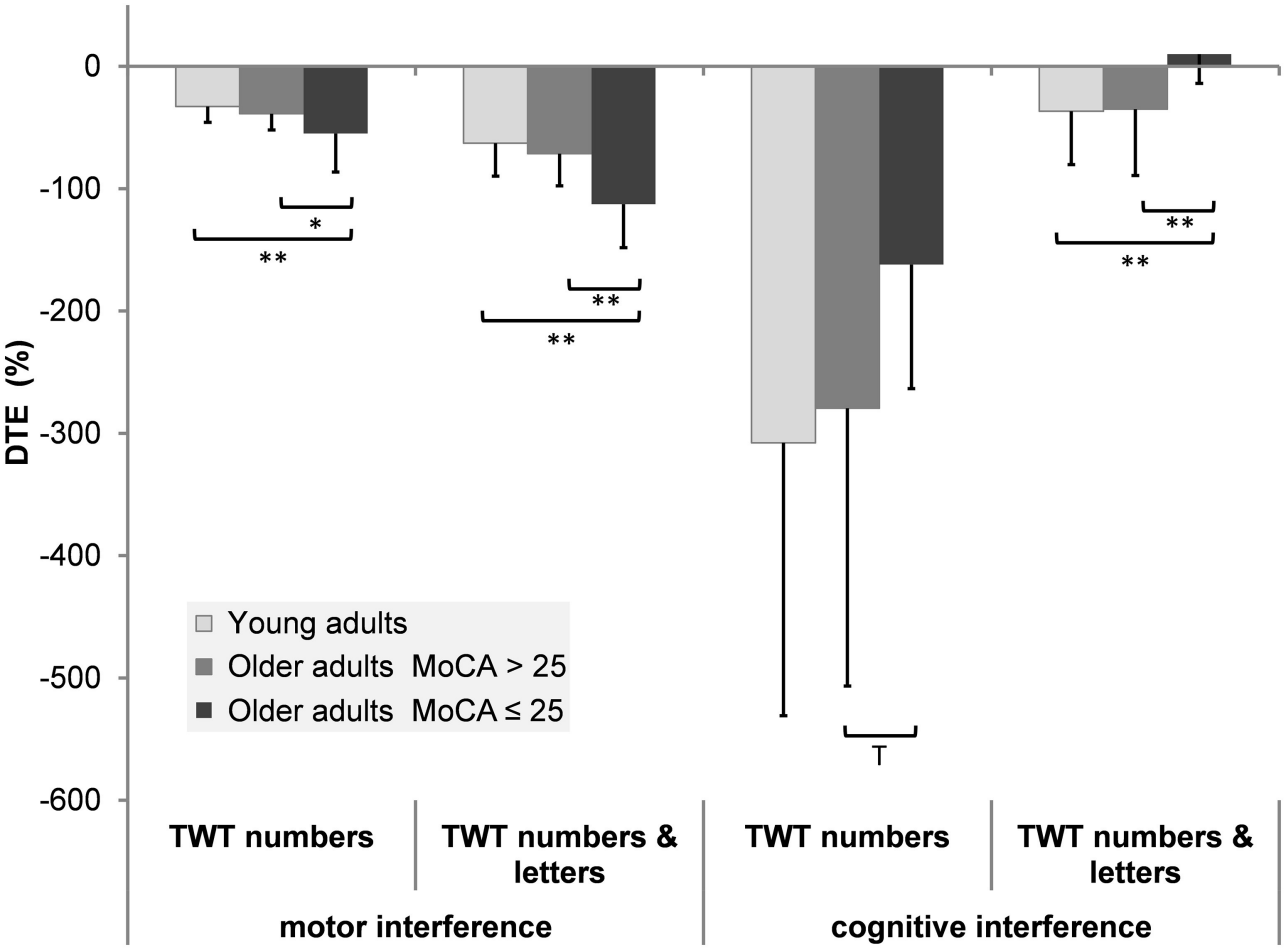
Patterns of cognitive-motor dual-task interference for the TWT by group (means and standard deviation) (^**^p < 0.01; ^*^p < 0.05; ^T^*p* < 0.10).

DTEs: Figure 3 shows that almost all individuals from all groups experienced mutual interference in the condition with low cognitive load, where both motor and cognitive performance declined under dual-task conditions. However, in the condition with high cognitive load only 68.6% in the group of younger adults, 69.6% in the group of healthy older adults, and 17.6% in the group with pMCI exhibited mutual interference, all other participants demonstrate DT costs on gait, but a DT benefit on cognition. Computations were made of the proportional changes between single and dual conditions of the TWT to check the motor and cognitive DTE, that is, the decrement in performance on either task relative to single-task performance. A 3 (group) × 2 (condition: low and high cognitive load) × 2 (CMI: cognitive vs. motor interference) ANOVA with repeated measures on the DTEs for times of the TWT was carried out, which showed a significant interaction for CMI × group, *F*_(2, 72)_ = 5.77, *p* < 0.001, η_*p*_^2^ = 0.138 was found: Older adults with pMCI exhibited larger effects on motor, but lower effects on cognitive DTEs than younger adults and older adults without pMCI. *Post-hoc* analysis confirmed significant differences for the motor and cognitive interference in the high load condition between the older adults with pMCI and both other groups (*p* < 0.05) (see Figure 4). Furthermore, there was a significant interaction for condition × CMI, *F*_(1, 72)_ = 104, *p* < 0.001, η_*p*_^2^ = 0.592, indicating higher motor costs in the high load condition (−43.0 ± 1.10) compared to the low load condition (−28.2 ± 1.02), but lower cognitive costs in in the high load condition (−20.6 ± 5.29) compared to the low load condition (−250 ± 24.6). The three TWT modalities provided an optimal differentiation between younger adults and older adults with and without pMCI (area under the curve > 0.9, *p* < 0.001), while discrimination between older adults with and without pMCI was only accurate for the TWT-3 (AUC > 0.8, *p* = 0.005). Furthermore, motor and cognitive DTEs for the more complex condition discriminate accurately between older adults with and without pMCI (see Table 3).

**Table 3:** Receiver operating characteristic curve statistics and cutoff thresholds for the TWT (raw scores, motor DTEs) to discriminate younger adults (Y), healthy older adults (HC), and older adults with probable MCI (pMCI).

**Variables**	**Group**	**n**	**Youden index**	**Sensitivity**	**Specificity**	**Score threshold**	**AUC**	***p***
TWT-1	Y vs. HC	42/24	0.74	95.8	78.6	25.31	0.941	< 0.001
	Y vs. pMCI	42/19	0.76	94.8	81.0	25.63	0.955	< 0.001
	HC vs. pMCI	24/19	0.22	47.4	75.0	33.71	0.612	0.211
TWT-2	Y vs. HC	42/24	0.86	100.0	85.7	36.09	0.975	< 0.001
	Y vs. pMCI	42/19	0.88	94.7	92.9	37.62	0.985	< 0.001
	HC vs. pMCI	24/19	0.44	52.6	91.7	52.63	0.704	0.020
TWT-3	Y vs. HC	42/24	0.83	100.0	83.3	41.29	0.953	< 0.001
	Y vs. pMCI	42/19	0.99	100.0	99.9	54.69	0.999	< 0.001
	HC vs. pMCI	24/19	0.67	100.0	66.67	57.18	0.860	< 0.001
DTEm TWT-2 – MS	Y vs. HC	37/24	0.41	70.8	70.3	−36.90	0.678	0.019
	Y vs. pMCI	37/19	0.49	79.0	70.3	−37.00	0.755	< 0.001
	HC vs. pMCI	24/19	0.31	47.0	83.3	−50.14	0.625	0.165
DTEm TWT-3 - MS	Y vs. HC	41/24	0.34	75.0	58.5	−59.96	0.646	0.043
	Y vs. pMCI	41/19	0.68	89.5	78.1	−73.70	0.890	< 0.001
	HC vs. pMCI	24/19	0.52	89.5	62.5	−73.70	0.794	< 0.001
DTEc_TWT-2 - MS	Y vs. HC	37/24	0.25	62.5	62.2	−495	0.602	0.183
	Y vs. pMCI	37/19	0.39	52.6	86.5	−283	0.687	0.017
	HC vs. pMCI	24/19	0.28	52.6	75.0	−281	0.596	0.283
DTEc TWT-3 - MS	Y vs. HC	41/24	0.19	33.3	85.4	−60.82	0.564	0.396
	Y vs. pMCI	41/19	0.54	68.4	85.4	−60.82	0.697	0.020
	HC vs. pMCI	24/19	0.43	68.4	75.0	−59.43	0.662	0.079

*AUROC, area under the receiver operating characteristic curve; TMT, Trail Making Test; TWT, Trail Walking Test; DTEm, Dual Task effects motor; MS, motor speed. For continuous variables cutoff thresholds were set at the optimum balance between sensitivity and specificity as determined by calculation of the Youden index. The threshold value for dichotomous variables was 1*.

## Discussion

This study was designed to evaluate the TWT as a potential and early detection tool for MCI. We therefore explored the effect of different types of cognitive tasks with different difficulties on cognitive-motor interferences in individuals with pMCI relative to healthy younger and older adults. For this reason, we used the TWT (Schott, 2015) as feasible, reliable and ecological valid dual task to better understand the relationship between cognitive and gross motor functions.

All participants performed slower under dual task conditions than under single task conditions, with the effect greater in older adults with pMCI. Increased cognitive task complexity resulted in greater slowing of trail walking and the difference between the three groups became more pronounced with increased task difficulty.

### Group differences for gait speed and errors

The performance for the three conditions of the TWT differ as expected from each other for the total as well as for the three performance groups. Our results are consistent with the findings of Pettersson et al. (2007) and Nascimbeni et al. (2015) who reported lower walking speed in MCI patient with respect to controls during single tasking. However, the differences between the older adults with and without pMCI were not statistically significant. Similarly to Lundin-Olsson et al. (1997) and Walshe et al. (2015) our results can thus show the effect that cognitive load has on gait. Recently, Walshe et al. (2015) reported large negative effects of dual-task interference on gait, but even more so when cognitive tasks are more executive in nature. The higher the cognitive load (targeting executive functions in the TWT-3), the longer it will take to complete the TWT, and the difference between the three groups became more pronounced. The TWT is even sensitive enough to distinguish between older adults with and without pMCI based on the duration needed and on the basis of the errors to complete the TWT. However, this is rather more pronounced in the condition with high cognitive load as evidenced by the interaction effect. This indicates that also the use of a gross motor task in a dual task paradigm may improve the early detection of MCI. Furthermore, we can confirm the strong interdependence between gait an cognition in older adults with cognitive impairment and dementia (Camicioli et al., 1998; van Iersel et al., 2004; Allan et al., 2005; Pettersson et al., 2005; Holtzer et al., 2006) and support the claim that the role of cognition is ultimately more pronounced in people with cognitive problems (Allali et al., 2015). This interdependency is explained by the fact that walking is a complex motor task, requiring executive functions (Hausdorff et al., 2005) and that walking as functional goal-oriented locomotion is not a merely automatic process, but requires higher-level cognitive input (Montero-Odasso et al., 2012; Schott et al., 2016). Especially in conditions in which people not only walk on a straight pathway, but in which they must orient themselves in space and have to change direction of movement as in the TWT (Schott, 2015).

Based on the calculated AUC we find fair (TWT-2, AUC = 0.704) and good (TWT-3, AUC = 0.860) power to differentiate between older adults with and without pMCI, however, we only observe a sufficient sensitivity in TWT-3 (100%). On the basis of these results and the exclusive account of the required times in the TWT, a differentiation between the two groups appears only appropriate through the TWT with higher cognitive load (TWT-3).

### Group differences for dual task effects

A closer look at the motor-cognitive interferences reveals that locomotion requires attention resources and that attention is a prerequisite for the effectiveness of normal walking in older adults (Smith et al., 2016). Overall, higher motor-related cognitive interferences rather than cognitive-related motor interferences were found. Higher cognitive-related motor DTEs were found for the task with higher cognitive load (the greater the cognitive load, the higher the motor interferences). The magnitude of the motor interference is comparable to the results of Salkovic et al. (2017), where subjects had to walk around a circle on the floor. The cognitive interferences, however, are many times higher in our dual task paradigm. This indicates that the TWT is much more demanding and claim much more cognitive capacity than walking a curved path while subtracting serial 7 s.

Furthermore, differences between the older adults with pMCI and both other groups were found for the motor DT effects in the low and high load condition with only poor (TWT-2, AUC = 0.625) diagnostic power in the low but good (TWT-3, AUC = 0.794) diagnostic power in the condition with high cognitive load. In contrast, higher motor related cognitive interferences were found for the task with lower cognitive load compared to the task with higher cognitive load. Differences between the older adults with pMCI and both other groups were only found for the cognitive interferences in the high load condition with just poor (TWT-3, AUC = 0.662) diagnostic power.

According to our results motor interferences in the TWT seem to supply an appropriate indicator for the early detection of MCI. Motor interferences seem sensitive enough to differentiate between older adults with and without pMCI (MacAulay et al., 2017). This supports previous findings showing a greater effect of DTEs on gait in MCI with respect to controls (Gillain et al., 2009; Maquet et al., 2010; Montero-Odasso et al., 2012; Muir et al., 2012) and is in contrast to the results of Nascimbeni et al. (2015) showing no DTE on gait in MCI. Overall, the effect is greater with the use of the high loaded cognitive task. This in turn is consistent with Nascimbeni et al. (2015) who showed that a counting backward task while walking may be too easy to detect early declines and therefore this task leads to a ceiling effect (e.g., Muir et al., 2012). The serial connection of numbers (1–15) while walking may as well involve cognitive processes (attention, visual scanning, motor speed and coordination) that are still preserved in the early stages of MCI (Nascimbeni et al., 2015), and therefore are too easy as secondary cognitive task. This is why we, based on the times needed, did not receive a good diagnostic power in the TWT-2 (AUC = 0.704), but a good to excellent diagnostic power with high sensitivity in the TWT-3 (AUC = 0.860). If, however, the motor interferences are used as basis for the assessment of the diagnostic power, only the condition with a high cognitive load is suitable (TWT-3, AUC = 0.794).

## General discussion

In the motor DT research, several factors have been suggested to account for different patterns in DT performance between young adults, older adults and older adults with MCI such as age, education, task priorization, and cognitive reserve (Stern, 2009; Schaefer, 2014; Belghali et al., 2017). One of the factors that has been implicated include the prioritization of posture/walking. In our study, the results indicate that by increasing the difficulty of the cognitive task, the resource allocation is increasingly directed toward the cognitive task and the motor task is neglected. All participants adapted a safe strategy and prioritized the motor task over the cognitive in the TWT (the so called “posture first” strategy). However, in older adults with pMCI they chose less to do so than in the other two groups. A shift of attention away from the walking task does not have the same ecological relevance like a shift in attention away from the cognitive task. Such a resource allocation strategy could lead to serious falls, especially in older adults, and even more so in individuals with attentional deficits, gait impairments, and an overall increased risk of falls (Montero-Odasso et al., 2012; Muir et al., 2012). The increased motor interferences in elderly individuals, especially those with MCI and increased attention limitations could explain the higher risk of falling (Liu-Ambrose et al., 2008; Mak et al., 2014). Indeed, all subjects who reported falls exhibited longer durations in all three conditions of the TWT. Thus, the TWT also could help - in addition to the early prediction of MCI—to predict future falls. This statement is, however, to be treated with caution because only marginal differences regarding the number of falls were found between the groups.

As compared with single-task standing or walking, the TWT effectively increases cognitive demand from condition to condition competing for common neural resources (Klingberg, 2000). This might represent an advantageous approach to examine how cognitive reserve (Stern, 2002) may be associated with better management of dual-task situations (Vallesi, 2016). Cognitive reserve refers to the potential to increase the efficiency and capacity of existing neural pathways and/or to recruit new pathways against the age-related and disease-related brain damage without developing cognitive deficits and/or clinical manifestations of disease (Belghali et al., 2017; Franzmeier et al., 2017; Gelfo et al., 2017). It is possible that our patients with pMCI have enough cognitive reserve to adequately pay attention to gait during a single task situation; however, in a DT condition there is not enough cognitive reserve to successfully perform an executive function task with high demands (Verghese et al., 2010; Perrochon et al., 2013). Similar to patients with Parkinson's disease, our subjects might have no excess cognitive resources to attribute to the cognitive task and therefore, perform worse than expected given their single task performance (Fuller et al., 2013). Fuller et al. (2013, p. 327) pointed out that this “may affect the everyday performance of simple tasks such as walking while navigating obstacles while contemplating everyday problems.”

The performance in the Trail-Walking-Test may reflect high-level visual processing and problem solving, aspects of executive functioning (Schott et al., 2016), which may be necessary when negotiating challenging terrain (Alexander et al., 2005). These functions play a crucial role in particular during cognitive aging and are strongly associated with frontal lobe performance (Dempster, 1992; Moscovitch and Winocur, 1992; Duncan et al., 1995; West, 1996). As previously indicated, numerous neuroscientific studies suggest that executive functions are limited due to age-related structural and functional changes in the integrity of the frontal lobe (frontal lobe hypothesis). In addition to cognitive processes age-related structural loss of prefrontal areas are correlated also with motor performance. Rosano et al. (2012) were able to show—based on imaging methods—that participants slow down their gait speed when walking due to a decrease in volume of the prefrontal areas. Similar results came from the InChanti study with 900 older dementia patients. The authors found a correlation between executive functions, operationalized by the Trail-Making-Test, and gait speed while walking through an obstacle course (see also Carlson et al., 1999; Bell-McGinty et al., 2002; Ble et al., 2005; Holtzer et al., 2006). So far, it can be stated that the frontal lobe is not only known for its role in executive functions such as attention and working memory (Scherder et al., 2007), but is also correlated to gait performance; in particular by its connection with the striatum (Pugh and Lipsitz, 2002) and the hippocampus (Bland and Oddie, 2001). Especially the hippocampus has a functional relationship with the prefrontal cortex (PFC) (frontal lobe) (Erickson and Barnes, 2003) and plays an important role in orientation of the body in space and on the integration of visual, vestibular and proprioceptive sensory and contextual information into spatial maps (Nutt et al., 1993; Scherder et al., 2007), which in turn is necessary for spatial orientation and navigation (Wolbers and Wiener, 2014). The degeneration of the hippocampus, which is a characteristic of MCI (Scheff et al., 2006) causes a disintegration of these information and thus leading to gait disturbances. Damage of the prefrontal cortex may cause executive dysfunctions, resulting also in gait disturbances (Yogev-Seligmann et al., 2008), e.g., by reducing the patients capacity to divide attention. Therefore, the positive relationship between walking and cognition might be explained by the functional relationship between the hippocampus and the prefrontal cortex (Scherder et al., 2007). In future studies the examination of neural correlates (EEG, fNIRS) are warrant to confirm this relationship and to understand the neurobiology of cognitive impairment and gait decline. Furthermore, although duration is a typical measurement in dual task research, other studies have shown that spatio-temporal features and joint angles are differentially related to dual-task performance (Montero-Odasso et al., 2009; Ponti et al., 2017). Hence, studies should include adaptive algorithms that quantify kinematic parameters such as step length, stride length, double support duration, head and body movements (Caldas et al., 2017). It is crucial that future research use a unified approach of physical performance measurements. Further efforts, based on a longitudinal study design, are also required to examine training effects on dual task effects, as well as different instructions focusing on time and/or accuracy (Schott et al., 2016).

## Conclusion

Our results suggest, similar to Allali et al. (2007) that the role of cognition in walking is ultimately more pronounced in people with pMCI and that dual task assessment shows promise as a potential marker for early detection of MCI. Therefore and on the basis of our results we recommend a gross-motor task such as functional goal-oriented locomotion in a more ecological valid environment as given in the TWT (Schott, 2015). Only when we are able to calculate DTE and evaluate performance in both, the motor and the cognitive task, we get useful insights into the possible preferential allocation of limited attention resources in MCI patients. Thus, it is possible to give individual recommendations for therapy (Schott, in press). This should emphasize why we need a common language and common standardized assessments between researchers in the field of Mobility and Cognition.

From our point of view and based on our results regarding sensitivity and specificity, we recommend the use of the TWT with the higher cognitive load as a new prodromal marker of dementia. It is an ecological valid dual task with excellent relative and absolute inter-trial reliability and has a high sensitivity and good - excellent diagnostic power to differentiate between older adults with and without pMCI (MacAulay et al., 2017).

Lastly, the examination of neural correlates (EEG, fNIRS) is absolutely essential to confirm the hypothesis that cognition and gross motor tasks share common neural networks.

## Author contributions

All authors listed have made a substantial, direct and intellectual contribution to the work, and approved it for publication.

### Conflict of interest statement

The authors declare that the research was conducted in the absence of any commercial or financial relationships that could be construed as a potential conflict of interest.

## Footnotes

^1^Adjusted MoCA score = raw MoCA score – 4.228 × [log_10_(100 – age) – 1.58] – 3.201 × [square root (years of education) – 3.25] (Santangelo et al., 2015).

## References

[B1] Aggarwal N. T. Wilson R. S. Beck T. L. Bienias J. L. Bennett D. A. (2006). Motor dysfunction in mild cognitive impairment and the risk of incident Alzheimer disease. Arch. Neurol. 63, 1763–1769. 10.1001/archneur.63.12.1763 17172617

[B2] Albert M. S. DeKosky S. T. Dickson D. Dubois B. Feldman H. H. Fox N. C.. (2011). The diagnosis of mild cognitive impairment due to Alzheimer's disease: recommendations from the national institute on aging-Alzheimer's association workgroups on diagnostic guidelines for Alzheimer's disease. Alzheimers Dement. 7, 270–279. 10.1016/j.jalz.2011.03.008 21514249 PMC3312027

[B3] Alexander N. B. Ashton-Miller J. A. Giordani B. Guire K. Schultz A. B. (2005). Age differences in timed accurate stepping with increasing cognitive and visual demand: a walking trail making test. J. Gerontol. A Biol. Sci. Med. Sci. 60, 1558–1562. 16424288 10.1093/gerona/60.12.1558

[B4] Allali G. Ayers E. I. Verghese J. (2015). Motoric cognitive risk syndrome subtypes and cognitive profiles. J. Gerontol. A Biol. Sci. Med. Sci. 71, 378–384. 10.1093/gerona/glv092 26248559 PMC5864158

[B5] Allali G. Kressig R. W. Assal F. Herrmann F. R. Dubost V. Beauchet O. (2007). Changes in gait while backward counting in demented older adults with frontal lobe dysfunction. Gait Posture 26, 572–576. 10.1016/j.gaitpost.2006.12.011 17344044

[B6] Allan L. M. Ballard C. G. Burn D. J. Kenny R. A. (2005). Prevalence and severity of gait disorders in Alzheimer's and non-Alzheimer's dementias. J. Am. Geriatr. Soc. 53, 1681–1687. 10.1111/j.1532-5415.2005.53552.x 16181166

[B7] Amboni M. Barone P. Iuppariello L. Lista I. Tranfaglia R. Fasano A. (2012). Gait patterns in Parkinsonian patients with or without mild cognitive impairment. Mov. Disord. 27, 1536–1543. 10.1002/mds.25165 23032876

[B8] American Psychiatric Association (2013). Diagnostic and Statistical Manual of Mental Disorders: DSM-5, 5th Edn. Washington, DC: American Psychiatric Association.

[B9] Atkinson G. Nevill A. M. (1998). Statistical methods for assessing measurement error (reliability) in variables relevant to sports medicine. Sports Med. 26, 217–238. 10.2165/00007256-199826040-00002 9820922

[B10] Bahureksa L. Najafi B. Saleh A. Sabbagh M. Coon D. Mohler M. J.. (2017). The impact of mild cognitive impairment on gait and balance: a systematic review and meta-analysis of studies using instrumented assessment. Gerontology 63, 67–83. 10.1159/000445831 27172932 PMC5107359

[B11] Barnes D. E. Yaffe K. (2011). The projected effect of risk factor reduction on Alzheimer's disease prevalence. Lancet. Neurol. 10, 819–828. 10.1016/S1474-4422(11)70072-2 21775213 PMC3647614

[B12] Beauchet O. Allali G. Launay C. Herrmann F. R. Annweiler C. (2013). Gait variability at fast-pace walking speed: a biomarker of mild cognitive impairment? J. Nutr. Health Aging 17, 235–239. 10.1007/s12603-012-0394-4 23459976 PMC12878871

[B13] Belghali M. Chastan N. Davenne D. Decker L. M. (2017). Improving dual-task walking paradigms to detect prodromal Parkinson's and Alzheimer's diseases. Front. Neurol. 8:207. 10.3389/fneur.2017.00207 28588547 PMC5438971

[B14] Bell-McGinty S. Podell K. Franzen M. Baird A. D. Williams M. J. (2002). Standard measures of executive function in predicting instrumental activities of daily living in older adults. Int. J. Geriatr. Psychiatry 17, 828–834. 10.1002/gps.646 12221656

[B15] Bland B. H. Oddie S. D. (2001). Theta band oscillation and synchrony in the hippocampal formation and associated structures: the case for its role in sensorimotor integration. Behav. Brain Res. 127, 119–136. 10.1016/S0166-4328(01)00358-8 11718888

[B16] Ble A. Volpato S. Zuliani G. Guralnik J. M. Bandinelli S. Lauretani F.. (2005). Executive function correlates with walking speed in older persons: the InCHIANTI study. J. Am. Geriatr. Soc. 53, 410–415. 10.1111/j.1532-5415.2005.53157.x 15743282

[B17] Bowie C. R. Harvey P. D. (2006). Administration and interpretation of the trail making test. Nat. Protoc. 1, 2277–2281. 10.1038/nprot.2006.390 17406468

[B18] Caldas R. Mundt M. Potthast W. Buarque de Lima Neto F. Markert B. (2017). A systematic review of gait analysis methods based on inertial sensors and adaptive algorithms. Gait Posture 57, 204–210. 10.1016/j.gaitpost.2017.06.019 28666178

[B19] Camicioli R. Bouchard T. Licis L. (2006). Dual-tasks and walking fast: relationship to extra-pyramidal signs in advanced Alzheimer disease. J. Neurol. Sci. 248, 205–209. 10.1016/j.jns.2006.05.013 16759668

[B20] Camicioli R. Howieson D. Oken B. Sexton G. Kaye J. (1998). Motor slowing precedes cognitive impairment in the oldest old. Neurology 50, 1496–1498. 10.1212/WNL.50.5.1496 9596020

[B21] Carlson M. C. Fried L. P. Xue Q. L. Bandeen-Roche K. Zeger S. L. Brandt J. (1999). Association between executive attention and physical functional performance in community-dwelling older women. J. Gerontol. B Psychol. Sci. Soc. Sci. 54, S262–S270. 10.1093/geronb/54B.5.S262 10542828

[B22] Cohen J. (1988). Statistical Power Analysis for the Behavioral Sciences. Hilsdale, NJ: Lawrence Earlbaum Associates.

[B23] Dempster F. N. (1992). The rise and fall of the inhibitory mechanism: toward a unified theory of cognitive development and aging. Dev. Rev. 12, 45–75. 10.1016/0273-2297(92)90003-K

[B24] Duffin J. T. Collins D. R. Coughlan T. O'Neill D. Roche R. A. Commins S. (2012). Subtle memory and attentional deficits revealed in an Irish stroke patient sample using domain-specific cognitive tasks. J. Clin. Exp. Neuropsychol. 34, 864–875. 10.1080/13803395.2012.690368 22643030

[B25] Duncan J. Burgess P. Emslie H. (1995). Fluid intelligence after frontal lobe lesions. Neuropsychologia 33, 261–268. 10.1016/0028-3932(94)00124-8 7791994

[B26] Erickson C. A. Barnes C. A. (2003). The neurobiology of memory changes in normal aging. Exp. Gerontol. 38, 61–69. 10.1016/S0531-5565(02)00160-2 12543262

[B27] Finlayson M. L. Peterson E. W. (2010). Falls, aging, and disability. Phys. Med. Rehabil. Clin. N. Am. 21, 357–373. 10.1016/j.pmr.2009.12.003 20494282

[B28] Fleiss J. L. (1999). Reliability of Measurement, in the Design and Analysis of Clinical Experiments. Hoboken, NJ: John Wiley and Sons, Inc.

[B29] Franzmeier N. Hartmann J. C. Taylor A. N. W. Araque Caballero M. Á. Simon-Vermot L. Buerger K.. (2017). Left frontal hub connectivity during memory performance supports reserve in aging and mild cognitive impairment. J. Alzheimer's Dis. 59, 1381–1392. 10.3233/JAD-170360 28731448 PMC5611800

[B30] Fuller R. L. Van Winkle E. P. Anderson K. E. Gruber-Baldini A. L. Hill T. Zampieri C.. (2013). Dual task performance in Parkinson's disease: a sensitive predictor of impairment and disability. Parkinsonism Relat. Disord. 19, 325–328. 10.1016/j.parkreldis.2012.11.011 23265679

[B31] Gaudino E. A. Geisler M. W. Squires N. K. (1995). Construct validity in the trail making test: what makes part B harder? J. Clin. Exp. Neuropsychol. 17, 529–535. 10.1080/01688639508405143 7593473

[B32] Gelfo F. Mandolesi L. Serra L. Sorrentino G. Caltagirone C. (2017). The neuroprotective effects of experience on cognitive functions: evidence from animal studies on the neurobiological bases of brain reserve. Neuroscience. [Epub ahead of print]. 10.1016/j.neuroscience.2017.07.065 28827089

[B33] Gillain S. Warzee E. Lekeu F. Wojtasik V. Maquet D. Croisier J.-L.. (2009). The value of instrumental gait analysis in elderly healthy, MCI or Alzheimer's disease subjects and a comparison with other clinical tests used in single and dual-task conditions. Ann. Phys. Rehabil. Med. 52, 453–474. 10.1016/j.rehab.2008.10.004 19525161

[B34] Hausdorff J. M. Yogev G. Springer S. Simon E. S. Giladi N. (2005). Walking is more like catching than tapping: gait in the elderly as a complex cognitive task. Exp. Brain Res. 164, 541–548. 10.1007/s00221-005-2280-3 15864565

[B35] Hilden J. Glasziou P. (1996). Regret graphs, diagnostic uncertainty and Youden's index. Stat. Med. 15, 969–986. 10.1002/(SICI)1097-0258(19960530)15:10<969::AID-SIM211>3.0.CO;2-9 8783436

[B36] Holtzer R. Verghese J. Xue X. Lipton R. B. (2006). Cognitive processes related to gait velocity: results from the einstein aging study. Neuropsychology 20, 215–223. 10.1037/0894-4105.20.2.215 16594782

[B37] Huy C. Schneider S. (2008). Instrument für die erfassung der physischen Aktivität bei personen im mittleren und höheren erwachsenenalter. Z. Gerontol. Geriatrie 41, 208–216. 10.1007/s00391-007-0474-y 18327696

[B38] Janoutov,á J. Šerý O. Hosák L. Janout V. (2015). Is mild cognitive impairment a precursor of Alzheimer's disease? Short review. Cent. Eur. J. Public Health 23:365 10.21101/cejph.a4414 26841152

[B39] Kenny R. A. Coen R. F. Frewen J. Donoghue O. A. Cronin H. Savva G. M. (2013). Normative values of cognitive and physical function in older adults: findings from the Irish longitudinal study on ageing. J. Am. Geriatr. Soc. 61(Suppl. 2), S279–S290. 10.1111/jgs.12195 23662720

[B40] Klingberg T. (2000). Limitations in information processing in the human brain: neuroimaging of dual task performance and working memory tasks. Prog. Brain Res. 126, 95–102. 10.1016/S0079-6123(00)26009-3 11105642

[B41] Lister J. J. Harrison Bush A. L. Andel R. Matthews C. Morgan D. Edwards J. D. (2016). Cortical auditory evoked responses of older adults with and without probable mild cognitive impairment. Clin. Neurophysiol. 127, 1279–1287. 10.1016/j.clinph.2015.11.007 26643153

[B42] Liu-Ambrose T. Donaldson M. G. Ahamed Y. Graf P. Cook W. L. Close J.. (2008). Otago home-based strength and balance retraining improves executive functioning in older fallers: a randomized controlled trial. J. Am. Geriatr. Soc. 56, 1821–1830. 10.1111/j.1532-5415.2008.01931.x 18795987

[B43] Lonie J. A. Tierney K. M. Ebmeier K. P. (2009). Screening for mild cognitive impairment: a systematic review. Int. J. Geriatr. Psychiatry 24, 902–915. 10.1002/gps.2208 19226524

[B44] Lowry K. A. Brach J. S. Nebes R. D. Studenski S. A. VanSwearingen J. M. (2012). Contributions of cognitive function to straight- and curved-path walking in older adults. Arch. Phys. Med. Rehabil. 93, 802–807. 10.1016/j.apmr.2011.12.007 22541307 PMC4878139

[B45] Lundin-Olsson L. Nyberg L. Gustafson Y. (1997). Stops walking when talking as a predictor of falls in elderly people. Lancet 349, 617. 10.1016/S0140-6736(97)24009-2 9057736

[B46] MacAulay R. K. Wagner M. T. Szeles D. Milano N. J. (2017). Improving sensitivity to detect mild cognitive impairment: cognitive load dual-task gait speed assessment. J. Int. Neuropsychol. Soci. 23, 493–501. 10.1017/S1355617717000261 28413999

[B47] Mak M. K. Wong A. Pang M. Y. (2014). Impaired executive function can predict recurrent falls in Parkinson's disease. Arch. Phys. Med. Rehabil. 95, 2390–2395. 10.1016/j.apmr.2014.08.006 25175162

[B48] Manckoundia P. Pfitzenmeyer P. d'Athis P. Dubost V. Mourey F. (2006). Impact of cognitive task on the posture of elderly subjects with Alzheimer's disease compared to healthy elderly subjects. Mov. Disord. 21, 236–241. 10.1002/mds.20649 16142775

[B49] Maquet D. Lekeu F. Warzee E. Gillain S. Wojtasik V. Salmon E.. (2010). Gait analysis in elderly adult patients with mild cognitive impairment and patients with mild Alzheimer's disease: simple versus dual task: a preliminary report. Clin. Physiol. Funct. Imaging 30, 51–56. 10.1111/j.1475-097X.2009.00903.x 19799614

[B50] Montero-Odasso M. Bergman H. Phillips N. A. Wong C. H. Sourial N. Chertkow H. (2009). Dual-tasking and gait in people with mild cognitive impairment. the effect of working memory. BMC Geriatr. 9:41. 10.1186/1471-2318-9-41 19723315 PMC2748075

[B51] Montero-Odasso M. Oteng-Amoako A. Speechley M. Gopaul K. Beauchet O. Annweiler C.. (2014). The motor signature of mild cognitive impairment: results from the gait and brain study. J. Gerontol. A Biol. Sci. Med. Sci. 69, 1415–1421. 10.1093/gerona/glu155 25182601 PMC4197903

[B52] Montero-Odasso M. Verghese J. Beauchet O. Hausdorff J. M. (2012). Gait and cognition: a complementary approach to understanding brain function and the risk of falling. J. Am. Geriatr. Soc. 60, 2127–2136. 10.1111/j.1532-5415.2012.04209.x 23110433 PMC3498517

[B53] Moscovitch M. Winocur G. (1992). The neuropsychology of memory and agingm, in The Handbook of Aging and Cognition, eds Craik F. I. M. Salthouse T. A. (Hillsdale, NJ: Erlbaum), 315–372.

[B54] Muir S. W. Speechley M. Wells J. Borrie M. Gopaul K. Montero-Odasso M. (2012). Gait assessment in mild cognitive impairment and Alzheimer's disease: the effect of dual-task challenges across the cognitive spectrum. Gait Posture 35, 96–100. 10.1016/j.gaitpost.2011.08.014 21940172

[B55] Nascimbeni A. Caruso S. Salatino A. Carenza M. Rigano M. Raviolo A.. (2015). Dual task-related gait changes in patients with mild cognitive impairment. Funct. Neurol. 30, 59–65. 26214028 PMC4520674

[B56] Nasreddine Z. S. Phillips N. A. Bédirian V. Charbonneau S. Whitehead V. Collin I.. (2005). The montreal cognitive assessment, MoCA: a brief screening tool for mild cognitive impairment. J. Am. Geriatr. Soc. 53, 695–699. 10.1111/j.1532-5415.2005.53221.x 15817019

[B57] Nutt J. G. Marsden C. D. Thompson P. D. (1993). Human walking and higher-level gait disorders, particularly in the elderly. Neurology 43, 268–268. 10.1212/WNL.43.2.268 8437689

[B58] Perrochon A. Kemoun G. Watelain E. Berthoz A. (2013). Walking Stroop carpet: an innovative dual-task concept for detecting cognitive impairment. Clin. Interv. Aging 8, 317–328. 10.2147/CIA.S38667 23682211 PMC3610448

[B59] Pettersson A. F. Olsson E. Wahlund L.-O. (2005). Motor function in subjects with mild cognitive impairment and early Alzheimer's disease. Dement. Geriatr. Cogn. Disord. 19, 299–304. 10.1159/000084555 15785030

[B60] Pettersson A. F. Olsson E. Wahlund L. O. (2007). Effect of divided attention on gait in subjects with and without cognitive impairment. J. Geriatr. Psychiatry Neurol. 20, 58–62. 10.1177/0891988706293528 17341772

[B61] Plummer P. Eskes G. (2015). Measuring treatment effects on dual-task performance: a framework for research and clinical practice. Front. Hum. Neurosci. 99:225 10.3389/fnhum.2015.00225 PMC4412054 25972801

[B62] Ponti M. Bet P. Oliveira C. L. Castro P. C. (2017). Better than counting seconds: identifying fallers among healthy elderly using fusion of accelerometer features and dual-task timed up and go. PLoS ONE 12:e0175559. 10.1371/journal.pone.0175559 28448509 PMC5407756

[B63] Pugh K. G. Lipsitz L. A. (2002). The microvascular frontal-subcortical syndrome of aging. Neurobiol. Aging 23, 421–431. 10.1016/S0197-4580(01)00319-0 11959405

[B64] Reitan R. M. (1955). The relationship of the trail making test to organic brain damage. J. Consult. Psychol. 19, 393–394. 10.1037/h0044509 13263471

[B65] Rosano C. Studenski S. A. Aizenstein H. J. Boudreau R. M. Longstreth W. T. Newman A. B. (2012). Slower gait, slower information processing and smaller prefrontal area in older adults. Age Ageing 41, 58–64. 10.1093/ageing/afr113 21965414 PMC3234076

[B66] Salkovic D. Hobert M. A. Bellut C. Funer F. Renno S. Haertner L.. (2017). Evidence for a selectively regulated prioritization shift depending on walking situations in older adults. Front. Aging Neurosci. 9:75. 10.3389/fnagi.2017.00075 28420979 PMC5378715

[B67] Santangelo G. Siciliano M. Pedone R. Vitale C. Falco F. Bisogno R.. (2015). Normative data for the montreal cognitive assessment in an italian population sample. Neurolog. Sci. 36, 585–591. 10.1007/s10072-014-1995-y 25380622

[B68] Schaefer S. (2014). The ecological approach to cognitive–motor dual-tasking: findings on the effects of expertise and age. Front. Psychol. 5:1167. 10.3389/fpsyg.2014.01167 25352820 PMC4196472

[B69] Scheff S. W. Price D. A. Schmitt F. A. Mufson E. J. (2006). Hippocampal synaptic loss in early Alzheimer's disease and mild cognitive impairment. Neurobiol. Aging 27, 1372–1384. 10.1016/j.neurobiolaging.2005.09.012 16289476

[B70] Scherder E. Eggermont L. Swaab D. van Heuvelen M. Kamsma Y. de Greef M.. (2007). Gait in ageing and associated dementias; its relationship with cognition. Neurosci. Biobehav. Rev. 31, 485–497. 10.1016/j.neubiorev.2006.11.007 17306372

[B71] Schott N. (2014). Reliability and validity of the German short version of the Activities specific Balance Confidence (ABC-D6) scale in older adults. Arch. Gerontol. Geriatr. 59, 272–279. 10.1016/j.archger.2014.05.003 24962236

[B72] Schott N. (2015). Trail walking test for assessment of motor cognitive interference in older adults: development and evaluation of the psychometric properties of the procedure. Z. Gerontol. Geriatrie 48, 722–733. 10.1007/s00391-015-0866-3 25801510

[B73] Schott N. (in press). Mobilitaet im Alter – Doppelaufgabentraining als Therapieform bei neurologischen Patienten. Swiss Sports Exerc. Med. 65.

[B74] Schott N. El-Rajab I. Klotzbier T. (2016). Cognitive-motor interference during fine and gross motor tasks in children with Developmental Coordination Disorder (DCD). Res. Develop. Disabil. 57, 136–148. 10.1016/j.ridd.2016.07.003 27428781

[B75] Sheridan P. L. Solomont J. Kowall N. Hausdorff J. M. (2003). Influence of executive function on locomotor function: divided attention increases gait variability in Alzheimer's disease. J. Am. Geriatr. Soc. 51, 1633–1637. 10.1046/j.1532-5415.2003.51516.x 14687395

[B76] Smith E. Cusack T. Blake C. (2016). The effect of a dual task on gait speed in community dwelling older adults: a systematic review and meta-analysis. Gait Posture 44, 250–258. 10.1016/j.gaitpost.2015.12.017 27004667

[B77] Stern Y. (2002). What is cognitive reserve? theory and research application of the reserve concept. J. Int. Neuropsychol. Soci. 8, 448–460. 10.1017/S1355617702813248 11939702

[B78] Stern Y. (2009). Cognitive reserve. Neuropsychologia 47, 2015–2028. 19467352 10.1016/j.neuropsychologia.2009.03.004 PMC2739591

[B79] Tabachnick B. G. Fidell L. S. (2013). Using Multivariate Statistics, 6th Edn. Boston, MA: Pearson.

[B80] Tseng B. Y. Cullum C. M. Zhang R. (2014). Older adults with amnestic mild cognitive impairment exhibit exacerbated gait slowing under dual-task challenges. Curr. Alzheimer Res. 11, 494–500. 10.2174/1567205011666140505110828 24801217 PMC4082490

[B81] Vallesi A. (2016). Dual-task costs in aging are predicted by formal education. Aging Clin. Exp. Res. 28, 959–964. 10.1007/s40520-015-0385-5 26006256 PMC5014893

[B82] van Iersel M. B. Hoefsloot W. Munneke M. Bloem B. R. Olde Rikkert M. G. (2004). Systematic review of quantitative clinical gait analysis in patients with dementia. Z. Gerontol. Geriatrie 37, 27–32. 10.1007/s00391-004-0176-7 14991293

[B83] Verghese J. Mahoney J. Ambrose A. F. Wang C. Holtzer R. (2010). Effect of cognitive remediation on gait in sedentary seniors. J. Gerontol. A Biol. Sci. Med. Sci. 65, 1338–1343. 10.1093/gerona/glq127 20643703

[B84] Verghese J. Robbins M. Holtzer R. Zimmerman M. Wang C. Xue X.. (2008). Gait dysfunction in mild cognitive impairment syndromes. J. Am. Geriatr. Soc. 56, 1244–1251. 10.1111/j.1532-5415.2008.01758.x 18482293 PMC2574944

[B85] Verghese J. Wang C. Lipton R. B. Holtzer R. (2013). Motoric cognitive risk syndrome and the risk of dementia. J. Gerontol. A Biol. Sci. Med. Sci. 68, 412–418. 10.1093/gerona/gls191 22987797 PMC3593614

[B86] Walshe E. A. Patterson M. R. Commins S. Roche R. A. (2015). Dual-task and electrophysiological markers of executive cognitive processing in older adult gait and fall-risk. Front. Hum. Neurosci. 9:200. 10.3389/fnhum.2015.00200 25941481 PMC4400911

[B87] Weir J. P. (2005). Quantifying test-retest reliability using the intraclass correlation coefficient and the SEM. J. Strength Cond. Res. 19:231. 10.1519/15184.1 15705040

[B88] West R. L. (1996). An application of prefrontal cortex function theory to cognitive aging. Psychol. Bull. 120:272. 10.1037/0033-2909.120.2.272 8831298

[B89] Wolbers T. Wiener J. M. (2014). Challenges for identifying the neural mechanisms that support spatial navigation: the impact of spatial scale. Front. Hum. Neurosci. 8:571. 10.3389/fnhum.2014.00571 25140139 PMC4121531

[B90] Yamada M. Ichihashi N. (2010). Predicting the probability of falls in community-dwelling elderly individuals using the trail-walking test. Environ. Health Prev. Med. 15, 386–391. 10.1007/s12199-010-0154-1 21432571 PMC2955901

[B91] Yogev-Seligmann G. Hausdorff J. M. Giladi N. (2008). The role of executive function and attention in gait. Mov. Disord. 23, 329–342. 10.1002/mds.21720 18058946 PMC2535903

